# Recurrent and *de novo* Glomerulonephritis After Kidney Transplantation

**DOI:** 10.3389/fimmu.2019.01944

**Published:** 2019-08-14

**Authors:** Wai H. Lim, Meena Shingde, Germaine Wong

**Affiliations:** ^1^Department of Renal Medicine, Sir Charles Gairdner Hospital, Perth, WA, Australia; ^2^School of Medicine, University of Western Australia, Perth, WA, Australia; ^3^NSW Health Pathology, Institute of Clinical Pathology and Medical Research, Westmead Hospital, Westmead, NSW, Australia; ^4^Sydney School of Public Health, University of Sydney, Sydney, NSW, Australia; ^5^Centre for Transplant and Renal Research, Westmead Hospital, Sydney, NSW, Australia; ^6^Centre for Kidney Research, The Children's Hospital at Westmead, Sydney, NSW, Australia

**Keywords:** recurrent disease, glomerulonephritis, kidney transplantation, recurrent glomerulonephritis, *de novo* glomerulonephritis, allograft failure

## Abstract

The prevalence, pathogenesis, predictors, and natural course of patients with recurrent glomerulonephritis (GN) occurring after kidney transplantation remains incompletely understood, including whether there are differences in the outcomes and advances in the treatment options of specific GN subtypes, including those with *de novo* GN. Consequently, the treatment options and approaches to recurrent disease are largely extrapolated from the general population, with responses to these treatments in those with recurrent or *de novo* GN post-transplantation poorly described. Given a greater understanding of the pathogenesis of GN and the development of novel treatment options, it is conceivable that these advances will result in an improved structure in the future management of patients with recurrent or *de novo* GN. This review focuses on the incidence, genetics, characteristics, clinical course, and risk of allograft failure of patients with recurrent or *de novo* GN after kidney transplantation, ascertaining potential disparities between “high risk” disease subtypes of IgA nephropathy, idiopathic membranous glomerulonephritis, focal segmental glomerulosclerosis, and membranoproliferative glomerulonephritis. We will examine in detail the management of patients with high risk GN, including the pre-transplant assessment, post-transplant monitoring, and the available treatment options for disease recurrence. Given the relative paucity of data of patients with recurrent and *de novo* GN after kidney transplantation, a global effort in collecting comprehensive in-depth data of patients with recurrent and *de novo* GN as well as novel trial design to test the efficacy of specific treatment strategy in large scale multicenter randomized controlled trials are essential to address the knowledge deficiency in this disease.

## Introduction

Primary glomerulonephritis (GN) continues to be one of the leading causes of end-stage kidney disease (ESKD) in the United States (US) and worldwide. According to the 2018 Australia and New Zealand Dialysis and Transplant (ANZDATA) registry report, GN as cause of ESKD accounted for 17% of incident treated ESKD patients (1). In the US, GN as cause of ESKD comprised of 7 and 13% of incident ESKD initiated on dialysis and have received kidney transplants, respectively; with similar proportion reported in the United Kingdom (2, 3). GN is a heterogeneous group of immunological kidney diseases with distinct histological subtypes, causes (primary vs. secondary) and clinical phenotypes, resulting in substantial differences in the prognosis after kidney transplantation in patients with dissimilar GN subtypes, including the risk of disease recurrence post-transplantation (4–7). Consequently, clinicians must provide sufficient information with regards to the risk of disease recurrence post-transplant, when considering the medical suitability of potential kidney transplant candidates with GN for kidney transplantation (4–6, 8–11).

Following kidney transplantation, recurrent or *de novo* GN in the renal allograft is an important cause of premature allograft failure (12). All GN subtypes can potentially recur after transplantation, with the prevalence of GN recurrence between 3 and 15%, particularly in patients with high risk subtypes of IgA nephropathy, idiopathic membranous GN, focal segmental glomerulosclerosis (FSGS), and membranoproliferative GN (MPGN) (4, 5, 9, 13, 14). Nevertheless, the incidence of *de novo* or recurrent GN after kidney transplantation is likely to be under-estimated because of the likelihood of selection bias (i.e., systematic differences in the selection and listing of ESKD patients with different GN subtypes), varying biopsy practices, ascertainment of the primary cause of ESKD, differing follow-up period, disparate clinical presentations ranging from asymptomatic urinary abnormalities to rapidly progressive GN, misclassification, and indication bias (where kidney biopsy may be carried out only for specific clinical indication and therefore may fail to identify asymptomatic incidental cases of early disease recurrence) and the competing risk of other causes of allograft failures. The risk of GN recurrence is typically directly related to incremental time post-transplant, with the majority of GN recurrence resulting in allograft failure occurring after 3–5 years post-transplant, although early recurrences can occur in patients with GN subtypes of MPGN and FSGS.

### Epidemiology, Pathogenesis, and Outcomes of GN Recurrence After Kidney Transplantation

The understanding and characterization of the incidence of GN recurrence occurring after kidney transplantation is largely confined to the availability of data from several large registries and single center reports from the US, Australia, and New Zealand (4, 5, 9, 13). The incidence of GN recurrence post-transplant varies according to GN subtypes and time post-transplantation. Of patients with ESKD secondary to primary GN, particularly FSGS and MPGN, there is a high risk of GN recurrence with a substantial proportion of patients with disease recurrence experiencing premature allograft failure.

### Recurrent IgA Nephropathy

Recurrent IgA nephropathy is relatively common, but typically occurs late post-transplant with cumulative incidence of disease recurrence at 15 years of 15% although the risk of disease recurrence may be reducing over time (Table 1) (5, 17). There is substantial variation between studies but likely to reflect disparate follow-up period, differing biopsy practices between centers and the relative benign presentations of the majority of patients (presenting with microscopic hematuria or evidence of IgA deposition in allograft biopsies). Following disease recurrence, up to 40% of patients with recurrent IgA nephropathy have been reported to lose their allografts, predominantly from disease recurrence (up to 60%) (5). Compared with other GN subtypes, the long-term allograft and patient outcomes of patients with IgA nephropathy are substantially better. In the US Renal Data System analysis of 32,131 patients with ESKD secondary to IgA nephropathy, the rates of all-cause mortality, overall allograft failure and death censored allograft failure were 1.2, 3.4, and 2.6 events/100-person-years, respectively; compared with respective 2.8, 6.7, and 5.1 events/100-person-years for patients with MPGN; 2.5, 6.1, and 4.4 events/100-person-years, respectively for patients with FSGS; and 3.1, 6.1, and 4.0 events/100-person-years, respectively for patients with idiopathic membranous GN (6). In this study, the proportion of allograft failure secondary to disease recurrence was 1.6% for those with IgA nephropathy, compared with 5.6, 2.7, and 3.3% for those with MPGN, FSGS, and idiopathic membranous GN, respectively (6). In contrast, data from the ANDATA registry showed that 5-year allograft survival following disease recurrence was similar between IgA nephropathy, idiopathic membranous GN, and FSGS, with the allograft survival of patients with MPGN substantially poorer compared to other GN subtypes (5).

**Table 1:** Prevalence, risk of allograft failure and clinical predictors of glomerulonephritis recurrence post-kidney transplantation.

	**Overall^**#**^**	**IgA Nephropathy**	**FSGS**	**Membranous GN**	**MPGN**
**Prevalence of GN recurrence**					
ANZDATA (1985–2014) (5)	10.3%	10% at 10 y, 15% at 15 y	9% at 10 y, 11% at 15 y	16% at 10 y, 18% at 15 y	16% at 10 y, 19% at 15 y
Mayo/Toronto* (4)	39.5% at 5 y	42% at 3 y, 51% at 5 y	31% at 3 y, 35% at 5 y	45% at 3 y, 55% at 5 y	41% at 3 y, 41% at 5 y
British Columbia (1990–2005) (9)	13% at 10 y, 18% at 15 y	15.4%	9.7%	10%	4.8% (type I MPGN only)
Korea (1995–2010)(11)	17.8%	14.8%	6.3%	0%	12.5%
France (single center) (15)	NR	36% at 10 y	NR	NR	NR
**Allograft failure following GN recurrence**					
ANZDATA (1985–2014) (5)	55%^♦^	58%^♦^	57%^♦^	59%^♦^	30%^♦^
RADR (1987–1996) (14)	5 y GS^θ^:40% (vs. 68% without)	Allograft failure 41%	Allograft failure 65%	Allograft failure 44%	Allograft failure 66%
Mayo/Toronto^#^ (4)	HR: 2.6 (1.9, 3.6)	HR: 3.4 (1.2, 9.7)	HR: 5.0 (2.4, 10.1)	HR 1.4 (0.3, 6.8)	HR 6.8 (2.7, 17.2)
British Columbia (1990–2005) (9)	HR: 7.5 (5.5, 10.2)	NR	NR	NR	NR
Korea (1995–2010) (11)	HR: 4.0 (1.7, 9.3)	NR	NR	NR	NR
**Clinical predictors of GN recurrence** (5, 9, 11, 15, 16)	Primary ESKD secondary to GN, male gender, younger age, non-white ethnicity, steroid-free	Younger age, steroid-free, early steroid-withdrawal, no induction therapy (ATG protective)	Younger age, rapid progression of initial ESKD	Presence (and titer) of anti-PLA2R autoantibody pre-transplant	C3-glomerulopathy subtypes, presence of monoclonal gammopathy, poor response to treatment and rapid progression to ESKD of native disease

♦ Denotes 5-year graft survival post-disease recurrence. Hazard ratio (HR) of death-censored allograft failure compared to kidney transplant recipients with same GN subtype but without disease recurrence post-transplant. *Denotes cumulative incidence.

# *May include recurrent and de novo GN. ^θ^ Denotes 5-year actuarial graft survival from time of transplant. GN, glomerulonephritis; HR, hazard ratio; MPGN, membrano-proliferative glomerulonephritis; FSGS, focal segmental glomerulosclerosis; ESKD, end-stage kidney disease; NR, not reported; ANZDATA, Australia and New Zealand Dialysis and Transplant registry; RADR, Renal Allograft Disease Registry; GS, graft survival; y, years; ATG, anti-thymocyte globulin*.

Several risk factors for disease recurrence have been described including younger age, recipients of zero-HLA-mismatched live-related donor kidneys, steroid-avoidance or early steroid-withdrawal immunosuppressive regimens, the non-use of induction therapy (whereas anti-thymocyte globulin [ATG] may be associated with a lower risk of recurrence), HLA allelic subtypes, crescentic (and rapidly progressive) IgA nephropathy in the native kidneys and shorter total ischemic time, but given these findings were identified in population cohort studies, it is difficult to ascertain the true causality of these risk factors for disease recurrence (5, 15, 16, 18–25). There are several molecules, including galactose-deficient IgA1 (Gd-IgA1), IgG anti-Gd-IgA1 antibodies, glycan-specific IgG antibodies, and soluble CD89 (an Fc receptor for IgA) that may be implicated in the pathogenesis of IgA nephropathy, the presence of which may portend a greater risk of disease progression and possibly disease recurrence post-transplant (26–32). There are several other non-specific serum and urine biomarkers that may predict the risk of disease recurrence after kidney transplantation, but the prognostic (and predictive) performance of many of these biomarkers have not been truly established or validated in independent population cohorts (Table 2). There are numerous other prognostic markers that have been investigated to predict disease progression of IgA nephropathy affecting the native kidneys, but the clinical relevance of these biomarkers in predicting disease recurrence post-transplant remains undetermined (51).

**Table 2:** Prognostic and predictive biomarkers for glomerulonephritis and recurrence of disease post-kidney transplant.

**Potential predictive biomarkers in GN subtypes**	**Clinical utility**	**Predict post-transplant recurrence**
**IgA nephropathy**		
Serum IgA level (33)	↑ Post-transplant predicts recurrence	Yes
*Serum galactose-deficient IgA1* (26)	↑ Pre-transplant predicts post-transplant recurrence	Yes
*Serum IgA-IgG complexes* (26)	↑ Pre-transplant predicts post-transplant recurrence	Yes
*Serum* IgA-sCD89 complexes (26)	↓ Pre-transplant predicts post-transplant recurrence	Yes
*Normalized Gd-IgA1-specific autoantibody* (34)	↑ Pre-transplant predicts post-transplant recurrence	Yes
Serum APRIL (35)	↑ Post-transplant predicts recurrence	Yes
^#^*Urine proteomics (SERPINA1, Transferrin, APOA4, and RBP4)* (36)	↑ Post-transplant predicts recurrence	Yes
**FSGS**		
*Serum* suPAR (37)	↑ Pre-transplant predicts post-transplant recurrence	Yes
*Urine suPAR* (38)	↑ Post-transplant predicts recurrence	Yes
Anti-CD40 autoAb (39)	↑ Pre-transplant predicts post-transplant recurrence	Yes
*Urine apolipoprotein A-1b* (40, 41)	↑ In relapses	No data
*A1AT* (42)	Differentiate from other causes	No data
CLC-1 (43)	↑ Recurrent disease	No data
*Anti-AT1R Ab*	↑ Pre-transplant predicts post-transplant recurrence	Yes
**Membranous GN**		
P*L*A2R *antibody* (44)	↑ Pre-transplant predicts post-transplant recurrence	Yes
*THSD7A autoantibody* (45, 46)	↑Primary membranous GN	No data
*Autoantigens of AR, SOD2*, α*ENO* (47)	↑Primary membranous GN	No data
**MPGN**		
Complements and C3NF (48–50)	Possible association with disease recurrence	Uncertain

# *Denotes abstract. GN, glomerulonephritis; FSGS, focal segmental glomerulosclerosis; MPGN, membranoproliferative GN; CLC-1, Cardiotrophin-like cytokine 1; THSD7A, Thrombospondin type 1 domain-containing 7A; AR, aldose reductase; αENO, α-enolase; AT1R Ab, angiotensin receptor II type 1 antibodies; PLA2R, phospholipase A2 receptor; C3NF, C3 nephritic factor; Gd, galactose-deficient; APRIL, a proliferation-inducing ligand; suPAR, soluble urokinase receptor; Ig, immunoglobulin*.

The optimal treatment of recurrent IgA nephropathy remains unknown and there are no current studies to suggest that alterations in immunosuppression will improve allograft outcomes (52). The current practice is to maintain (or change to) a calcineruin-inhibitor (CNI) and corticosteroids-based immunosuppressive regimen in addition to anti-proteinuric treatments, although the optimal dosing/target therapeutic CNI level or specific CNI type in the treatment of those with recurrent disease remains unknown (Table 3). In cases of crescentic rapidly progressive IgA nephropathy, more aggressive immunosuppression (e.g., cyclophosphamide or rituximab) may be considered but this is largely unproven and unlikely to successfully reverse the disease process (53–56). The potential benefit of tonsillectomy in disease recurrence has been limited to case reports and therefore cannot be recommended as a treatment option for patients with recurrent IgA nephropathy (57, 58).

**Table 3:** Proposed management options for recurrent glomerulonephritis.

	**Initial treatment**	**Other options**	**Trials^**#**^**
Recurrent IgA Nephropathy	Anti-proteinuric CNI + steroid*	Alkylating agents (crescentic) (Tonsillectomy)	Induction (ATG vs. basiliximab)
Recurrent FSGS	Anti-proteinuric Plasmapheresis ± rituximab CNI	Ofatumumab Abatecept/belatacept	Pre-emptive rituximab Acthar Bleselumab Total lymphoid irradiation
Recurrent idiopathic membranous GN	Anti-proteinuric CNI Rituximab (antibody positive)	Rituximab (antibody negative) Bortezomib Alkylating agents	
Recurrent MPGN	Anti-proteinuric Treat monoclonal gammopathy (if present)	Eculizumab if C3 glomerulopathy Plasmapheresis and Immunosuppression (alkylating agent, rituximab) if immune complex MPGN	

* *Optimal dose or combination of CNI type and corticosteroids unknown*.

# *Trials (registered in progress/recruiting or not yet recruiting) as searched in: https://clinicaltrials.gov. GN, glomerulonephritis; CNI, calcineurin-inhibitor; FSGS, focal segmental glomerulosclerosis; MPGN, membranoproliferative GN; ATG, anti-thymocyte glbulin*.

### Recurrent Primary FSGS

Up to 1 in 3 patients with primary FSGS will experience disease recurrence after kidney transplantation, with the risk of allograft failure (predominantly from GN recurrence) 5-times the risk compared to those without disease recurrence (Table 1) (4, 5). In an ANZDATA registry analysis comprising of 736 first kidney transplant recipients with biopsy-proven primary FSGS, 10% of patients experienced disease recurrence, with disease recurrence associated with substantially poorer 5-year allograft survival of 52% (95% confidence interval [95%CI], 40%, 63%), compared with 83% (95%CI 79%, 86%) in those without recurrent disease (*p* < 0.001) (59). However, the true incidence of recurrent disease remains unknown as secondary forms of FSGS can occur late post-transplant, resulting in difficulties in differentiating primary from secondary forms.

In contrast to patients with primary FSGS, familial FSGS in adults, comprising of those with mutations of podocin or structural podocyte proteins [e.g., NPHS2, including those with homozygous or compound heterozygous mutations in podocin or the p.R229Q variant; slit diaphragm-associated transient receptor potential channel C6 [TRPC6] gain-of-function mutation] and apolipoprotein L-1 genotype have low to no risk (<3%) of disease recurrence post-transplant suggesting the relative importance of genetic testing in the evaluation of a subset of patients with adult-onset FSGS for transplantation (60–67). Other than a known family history of chronic kidney disease (which may suggest an autosomal dominant inheritance), many of the genetic mutations associated with adult-onset FSGS have an incomplete penetrance and therefore, the identification of patients with familial FSGS remains challenging (68). Clinicians should consider undertaking genetic screening for patients with adult-onset FSGS when there is uncertainty regarding the likelihood of primary (atypical clinical/pathological features or poor response to immunosuppressive treatment) and secondary (no obvious causes identified) FSGS or when there is a clear family history of FSGS (69, 70). Taking into consideration the cost associated with genetic screening in all patients with adult-onset FSGS, it may not be cost-effective to screen all patients (even with the above criteria) and therefore, it may be more practical to consider screening for patients with a clear family history of FSGS or those with a potential live-related donor for transplantation, including undertaking genetic screening of the donors for the same genetic mutations (if present in the potential recipients). In the absence of genetic mutation in the potential recipients, genetic screening of live-related donors is not currently recommended, although cases of live donors developing FSGS post-donation have been reported (66, 71). A shared-decision approach between clinicians and patients regarding the clinical rationale for genetic screening for patients and potential live-related donors should be considered, balancing between the cost, the clinical utility of the information in the current/future medical management of these patients pre- and post-transplant and the implications for prognostication (post-transplant) and appropriateness of genetic counseling. A similar practical approach to genetic testing may be considered for pediatric patients with FSGS, including those with idiopathic steroid-resistant nephrotic syndrome with FSGS pathology. In a Spanish cohort of 98 children or adolescent patients with FSGS (<18 years at presentation), none of the 7 patients (presented with steroid-resistant nephrotic syndrome and documented NPHS2 mutation) who had received a kidney transplant experienced disease recurrence, suggesting that similar to the adult population, there is a low risk of FSGS recurrence in pediatric patients with genetic FSGS post-transplant (72, 73). There are several other risk factors for disease recurrence in patient with primary FSGS that have been identified, although these are primarily non-specific clinical parameters including younger age at presentation, recipients of live-donor kidneys, non-white ethnicity, severe manifestations of disease at presentation, rapid progression to ESKD, and prior allograft failure from disease recurrence (5, 59, 74).

The pathogenesis of disease recurrence in patients with primary FSGS remains unclear, with no studies confirming the presence of circulating permeability factor(s) causing podocyte injury instigating early disease recurrence. A pathogenic role of the circulating serum soluble urokinase receptor (suPAR) has been proposed, by activating podocyte β(3) integrin resulting in effacement of foot processes and proteinuria, which may contribute to the development of primary FSGS. In two cohorts of pediatric and adult patients with primary FSGS from North America [*n* = 70, North America–based FSGS clinical trial [FSGS-CT] cohort] (75) and Europe (*n* = 94, PodoNet Registry cohort) (76), serum suPAR level (threshold of 3,000 pg/ml) was elevated in over 50% of patients (84% FSGS-CT and 55% PoDoNet cohorts), compared with 6% of age- and sex-matched healthy controls (*n* = 150) (77, 78). Interestingly, in the European cohort, serum suPAR level was higher in patients with familial FSGS compared to those with non-genetic primary FSGS, but this finding will need to be validated in other cohorts. In addition, reduction of serum suPAR level with immunosuppressive treatment was associated with a greater likelihood of achieving clinical remission, raising the possibility that suPAR may be involved in the pathogenesis of this disease (78). However, this patho-physiological link remains debatable (37, 79–81). Furthermore, serum suPAR level can also be non-specifically elevated in other pathological processes including inflammation and infection and has been shown to be an independent prognostic biomarker in predicting future risk of CVD and mortality in the general population (82, 83).

Nevertheless, the diagnostic test accuracy of suPAR in differentiating primary FSGS from other proteinuric diseases or to predict disease recurrence after transplantation remains suboptimal (84, 85) and consequently, the clinical utility of routinely monitoring suPAR levels post-transplant to predict those at risk of disease recurrence remains poorly defined. Other potential biomarkers including urine suPAR, Anti-CD40 autoantibody, and angiotensin receptor II type 1 (AT1R) antibody appear promising but further studies are required to determine the accuracy of these prognostic biomarkers in predicting disease recurrence (Table 2). Close monitoring for proteinuria in high risk patients, with regular checks of urine protein/creatinine ratio or self-check urine dip-stick in the first 3 months post-transplant are recommended, and proceeding to a kidney biopsy (tissues should be sent for electron microscopy to detect early effacement of foot processes) if there is persistent or increasing proteinuria (86).

The management of patients with recurrent primary FSGS remains challenging, with treatment strategies informed predominantly by small case series (Table 3). Plasmapheresis is often preferred and recommended (in the American Society for Apheresis guidelines) in the treatment of primary FSGS recurrence in the allograft of both pediatric and adult patients (87–89). The efficacy of adjunctive therapy including rituximab and CNI such as cyclosporine remains uncertain. The case report of the efficacy of rituximab in ameliorating early primary FSGS recurrence post-transplant in a child with post-transplant lymphoma had generated considerable interest and suggested that B cells may have a role in the pathogenesis of disease recurrence in a subgroup of patients with primary FSGS (90). Recent research showed that rituximab binds sphingomyelin phosphodiesterase acid-like 3b (SMPDL-3b) protein, resulting in preservation of podocyte SMPDL-3b expression, preventing podocyte apoptosis, and maintaining the integrity of podocyte actin cytoskeleton, therefore highlighting the biological rationale of this therapy in patients with FSGS independent of its effect on B cells (91). Given the lack of convincing evidence to suggest B cells is directly implicated in the pathogenesis of primary and recurrent FSGS (despite one small biopsy series showing higher number of glomerular B cells in patients with FSGS) (92), the mechanism by which rituximab may be effective in reducing proteinuria in patients with recurrent FSGS may be through its effect on podocyte function (91, 93). CNI such as cyclosporine, through the inhibitory effect on T cell function and stabilization effect on actin cytoskeleton in kidney podocytes, has been shown to induce clinical remission in patients with recurrent FSGS. In patients already maintained on CNI and have experienced disease recurrence post-transplant, there is no data to suggest that changing to an alternative CNI (e.g., from cyclosporine to tacrolimus or from tacrolimus to cyclosporine) will be effective. However, a switch to CNI if patients were on mammalian target of rapamycin [mTOR]-inhibitor is advocated, given that mTOR-inhibitor, especially at higher doses have been associated with the development of *de novo* FSGS (94–96). What remains unknown is whether plasmapheresis is always necessary and whether rituximab should be considered as first line therapy for disease recurrence or reserved only for cases of recurrence refractory to plasmapheresis (97).

The roles of pre-emptive plasmapheresis (pre and/or post-transplantation), immunoadsorption therapy, and other novel options such as ofatumumab or B7-1 blockers (abatacept and belatacept) to prevent disease recurrence or to treat recurrence appear promising but the efficacy of these treatments remain debatable and not always consistent or supported in subsequent studies (98–105). Ofatumumab, an anti-CD20 monoclonal antibody that induces profound B cell depletion appears promising in the prevention (*n* = 1) and treatment (*n* = 2, achieved partial remission) of recurrent FSGS occurring post-kidney transplant but this needs to be confirmed in large studies (103, 106, 107). The initial treatment success surrounding the efficacy of abatacept, an inhibitor of B7-1 co-stimulatory molecule in achieving clinical remission in 5 patients with FSGS (4 patients with rituximab-resistant recurrent FSGS post-kidney transplant and 1 patient with steroid-resistant primary FSGS; all with positive B7-1 immunostaining of podocytes) has not been corroborated in other cohorts (100). Five subsequent studies showed that abatacept or belatacept was not effective in the treatment of 23 patients with FSGS recurrence post-kidney transplant, although the majority of the patients did not have positive podocyte B7-1 expression (101, 102, 105, 108, 109).

In a retrospective historical-control study of 26 pediatric patients with FSGS, prophylactic pre- and post-transplant plasmapheresis did not prevent FSGS recurrence compared to those who did not undergo similar prophylactic treatment (110). Several pilot studies evaluating the efficacy of an intensive and prolonged plasmapheresis with and without high-dose corticosteroids and maintenance CNI therapy or rituximab (2 studies, *n* = 22 adult patients with primary FSGS recurrence [2 in native kidneys], up to 15 months of treatment) or immunoadsorption (*n* = 12 pediatric patients with early FSGS recurrence) may be effective in achieving partial or complete remission but given the retrospective nature of these studies in a small number of patients, these and other treatment regimens will need further appraisal in randomized controlled trials to ascertain the optimal management in preventing or treatment of FSGS recurrence (104, 111, 112). A practical approach in the management of FSGS recurrence post-transplantation should initially include plasmapheresis, maximizing anti-proteinuric therapy, and converting to a CNI-based immunosuppressive regimen (where possible), with B cell depletion (rituximab) considered as adjunctive treatment or in resistant cases (Table 3). There is currently insufficient data to suggest that the pre-emptive use of plasmapheresis ± rituximab will reduce the risk of disease recurrence post-transplant.

### Recurrent Primary MPGN

Disease recurrence post-transplant from primary MPGN is relatively common, with over 50% of recurrence occurring within the first 24 months post-transplant (Table 1) (5, 13, 48, 49, 113, 114). In patients who had experienced disease recurrence, the risk of allograft failure is relatively high with 5-year allograft survival post-disease recurrence of only 30% (5). The introduction of a new classification of MPGN, which considers the differences in the pathogenesis and histological findings of MPGN subtypes (i.e., immune complex-mediated MPGN and complement-mediated MPGN), has enabled a more accurate assessment of the nature and course of the disease, including the risk of disease recurrence after kidney transplantation (115–117). Immune-complex mediated MPGN is characterized by the glomerular deposition of polyclonal or monoclonal immunoglobulins (Ig), whereas C3 glomerulopathy [comprising of C3GN and dense deposit disease (DDD)] is characterized by the glomerular deposition of C3 in the absence of Ig deposition (115, 117, 118). The pattern of glomerular Ig and complement product deposition may help to differentiate between the MPGN subtypes and has also provided much needed insights and information on the pathogenesis of the different disease processes (4, 116). Even though DDD (previously known as MPGN type II) and C3GN are distinctive diseases, the clinical course, pathogenesis and histological features of these two diseases may be similar. There is a high rate of post-transplant recurrence for both C3GN and DDD, with over 50% of patients with disease recurrence reported to experience allograft failure, although the number of patients in these studies was relatively small (119, 120). The timing and clinical presentations of patients with C3GN and DDD may be dissimilar, with DDD more likely to recur later post-transplant and often associated with no clinical manifestations other than allograft dysfunction. C3GN and DDD are characterized by the presence of strong glomerular staining for C3 and electron deposits on electron microscopy, but these diseases are potentially morphologically distinguishable by the nature and ultrastructural characteristics of these electron dense deposits (115, 121–123). The predominance of C3 deposition suggest the presence of dysregulated alternate complement cascade, resulting in the amplification and subsequent overproduction of C3 and related products of the terminal complement cascade (124). The exact cause of the complement dysregulation remains uncertain, although genetic mutations (e.g., presence of H402 and V62 alleles of Factor H, mutations of Factor H, and I genes) or autoantibodies (e.g., C3 or C4 nephritic factor directed against C3 convertase or Factor H autoantibodies) resulting in dysfunctional complement regulatory proteins and therefore uncontrolled amplification of the C3 protein have been implicated in the pathogenesis of this disease (125–129). In a case series of 21 kidney transplant recipients with C3GN as the cause of ESKD, monoclonal gammopathy was present in 3 of 14 (21%) patients who had experienced disease recurrence (suggesting the involvement of classical complement pathway), which was associated with a more rapid rate of disease recurrence (median time to recurrence 4 vs. 43 months, respectively) and allograft failure compared to those without monoclonal gammopathy (119).

**Figure 1:**
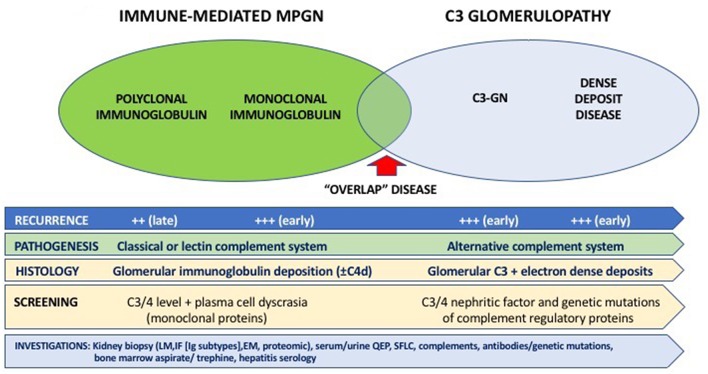
Overview of the classification, pathogenesis, characteristics, and diagnostic assessment of membranoproliferative glomerulonephritis (MPGN).

**Figure 2:**
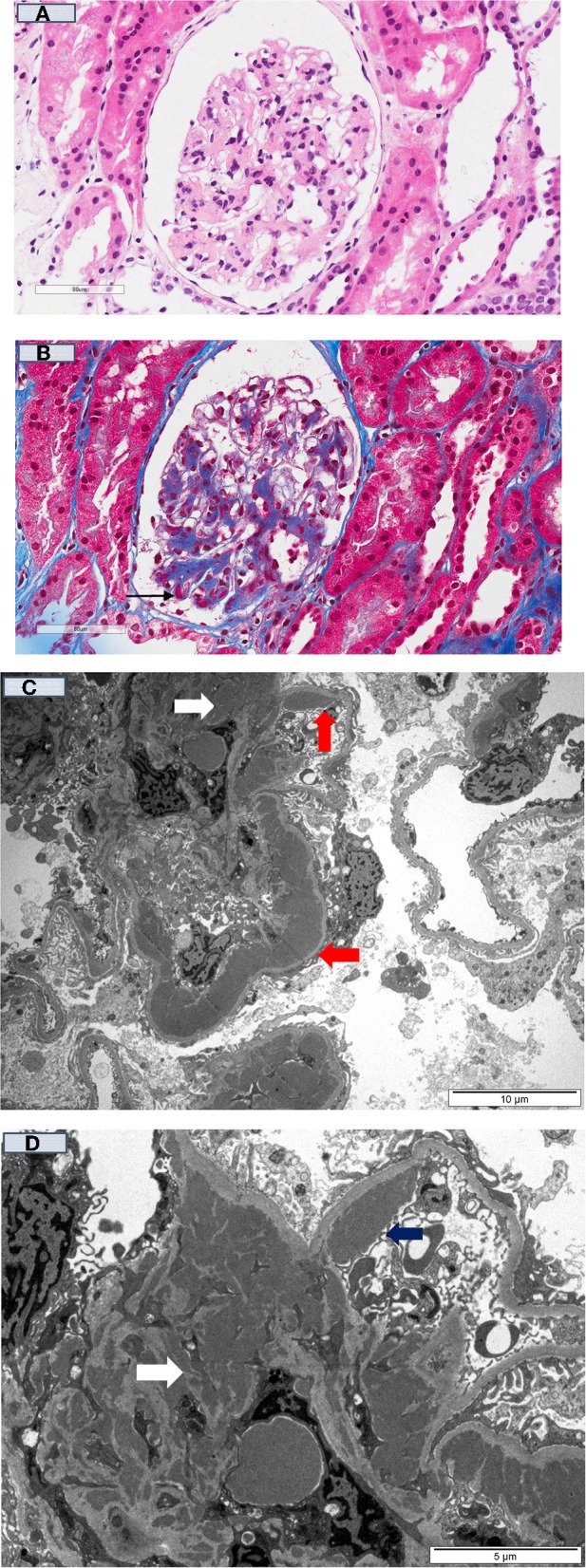
Kidney transplant recipient who had developed recurrent MPGN 12-months post-transplant. **(A)** [H & E (40x)]: Kidney allograft biopsy showed mesangial matrix expansion and a few thick peripheral capillary loops; **(B)** [Gomori trichrome stain (40X)] showed the thick peripheral capillary loops containing dense eosinophilic deposits (black arrow); and **(C)** (10 μm magnification), and **(D)** (5 μm magnification) showed numerous electron-dense deposits within the mesangium (white arrow) and large band-like intramembranous (red arrow) and subendothelial (blue arrow) electron-dense deposits within the thick peripheral capillary loops (Electron Microscopy).

For immune-mediated MPGN, the patterns and types of Ig deposits may have diagnostic and prognostic significance. The presence of serum monoclonal proteins (48, 49), with and without low complement levels (±glomerular C4d deposition) (130), implying activation of the complement cascade was associated with a less favorable clinical course post-transplant, with higher risk of recurrence and disease progression following recurrence. Similarly, the presence of glomerular monoclonal Ig deposits (typically IgG3κ and IgG3λ, but IgG2λ has been reported) was associated with poorer prognosis, characterized by early disease recurrence and substantially greater risk of premature allograft failure following disease recurrence (131–134). Up to 70% of patients with immune-mediated GN and monoclonal deposits have no evidence of plasma cell dyscrasia (i.e., absence of serum and urine monoclonal Ig proteins or evidence of plasma cell dyscrasia in the bone marrow), whereas in the remaining 30% of patients, patients often have detectable elevated monoclonal proteins without fulfilling the criteria for multiple myeloma (often termed “monoclonal gammopathies of renal significance”) (135–137). It is important to note that these disease processes on occasions have overlapping clinical and histological features (e.g., monoclonal gammopathy may be present in both immune complex-mediated and complement-mediated MPGN) and clinicians should consider undertaking a panel of investigations for all patients with MPGN being assessed for transplantation (Figure 1). Despite the advances in the current understanding of the pathogenesis and risk of disease recurrence in patients with MPGN, there continues to be residual uncertainties as to the relationships between patient and disease characteristics and the risk of disease recurrence and longer-term prognosis. Clinicians should be alerted of the need to undertake pre-transplant screening for monoclonal gammopathy ± hematology review in patients with MPGN, while ensuring close monitoring post-transplant for signs of disease recurrence. Open discussion to ensure patients with MPGN are counseled appropriately such that they are cognizant of the risk of disease recurrence post-transplant, weighing between this risk of premature allograft failure (if disease recurs) against that associated with remaining on dialysis (138). Figures 2A–D show the light microscopy and ultrastructural features of a kidney transplant recipient who had developed asymptomatic MPGN recurrence 12-months post-transplant, with similar patterns of electron dense deposits as the primary disease.

The approach to treatment for recurrent disease is not well-established, limited to case series of successful treatment with the use of plasmapheresis and other immunosuppressive agents including cyclophosphamide, eculizumab, and rituximab (139–142). However, the optimal treatment strategy remains unknown (100, 101, 143, 144). In a case series of 7 patients with C3 glomerulopathy treated with eculizumab for a duration between 3 and 28 months (*n* = 5 with C3GN and *n* = 2 with DDD), 3 (all with C3GN) of the 7 patients had received a kidney transplant. Of these 3 patients, 2 had experienced disease recurrence between 3 and 48 months post-transplant, and 1 patient had developed *de novo* C3GN at 135 months post-transplant (original ESKD attributed to FSGS). The initiation of eculizumab in these 3 patients resulted in stabilization of kidney function and proteinuria in 2 patients, with no response in 1 patient and treatment with eculizumab was subsequently discontinued (139). Other reports comprising of 6 pediatric patients with MPGN I, C3GN, or DDD (*n* = 5 native kidneys and *n* = 1 with recurrence of C3 glomerulopathy post-kidney transplant) showed a modest benefit of eculizumab in reducing proteinuria and stabilization of kidney function suggesting a potential therapeutic role of eculizumab in a subgroup of patients (145, 146). Nevertheless, given the heterogeneity in the use and varying treatment responses to eculizumab in these cases (and small number of cases of post-transplant recurrence), larger studies are required to ascertain the true benefits of eculizumab in the treatment of disease recurrence post-transplant. The use of plasmapheresis and/or rituximab in the treatment of primary MPGN recurrence post-transplant has been limited to a small number of cases (*n* = 9), with modest response observed in half the cases. In one patient who had developed recurrent crescentic DDD and had failed treatment with rituximab and plasmapheresis, the introduction of eculizumab therapy led to discernible clinical and biochemical responses (119, 140, 141, 147, 148). In a retrospective Spanish cohort study of 60 patients with C3GN (affecting native kidneys), patients who were maintained on immunosuppressive treatment, particularly corticosteroids and mycophenolate were more likely to achieve clinical remission compared to untreated patients or those on other immunosuppressive regimens (149). However, the relevance of this observation in the treatment of disease recurrence post-transplantation is unknown with no current data to support a dose increase of mycophenolate or a change from an alternative regimen to a mycophenolate-based immunosuppressive regimen.

The new classification and current knowledge of MPGN may assist clinicians to adopt a personalized treatment strategy according to the most likely pathogenesis of the disease process (e.g., consideration of plasmapheresis and anti-B cell therapy for immune complex-mediated GN or those with monoclonal gammopathy or to consider eculizumab for C3 glomerulopathy) and the availability of more cases with longer-term follow-up data will be essential in determining the most appropriate treatment options for patients who have development recurrent disease post-transplant (Table 3).

### Recurrent Idiopathic Membranous GN

The discoveries of major podocyte antigens and the pathogenic autoantibody against the podocyte antigen phospholipase A2 receptor (PLA2R) have led to breakthroughs in the understanding, management, and treatment of patients with idiopathic membranous GN (150, 151). The ability to test for the presence of anti-PLA2R autoantibody has resulted in improved recognition and differentiating primary vs. secondary membranous GN, as well as assisting clinicians in the management of patients pre and post-transplantation, identifying those patients at high risk of post-transplant disease recurrence that may benefit from more intensive monitoring post-transplant as well as monitoring response to treatment (44, 152, 153). Nevertheless, the absence of PLA2R autoantibody does not definitively exclude cases of idiopathic membranous GN (45, 154). The diagnostic test accuracy of circulating anti-PLA2R autoantibody in differentiating primary from secondary membranous GN is acceptable, with reported test sensitivity of 65% (63–67%), specificity of 97% (97–98%), positive likelihood ratio of 15.65 (9.95–24.62), and negative likelihood ratio of 0.37 (0.32–0.42) (155). Similar test performance accuracy is shown for the positive glomerular staining of PLA2R antigen (155, 156). A second antibody specific for the autoantigen thrombospondin type 1 domain–containing 7A (THSD7A) has been detected in up to 5% of patients with idiopathic membranous GN, typically in those who were seronegative for the PLA2R autoantibody (<15% cases) (45). Nevertheless, up to 20% of patients with idiopathic membranous GN do not have detectable autoantibodies to PLA2R or THSD7A, suggesting the possibility that unidentified autoantibodies targeting other auto-antigens may be contributing (47, 157).

**Figure 3:**
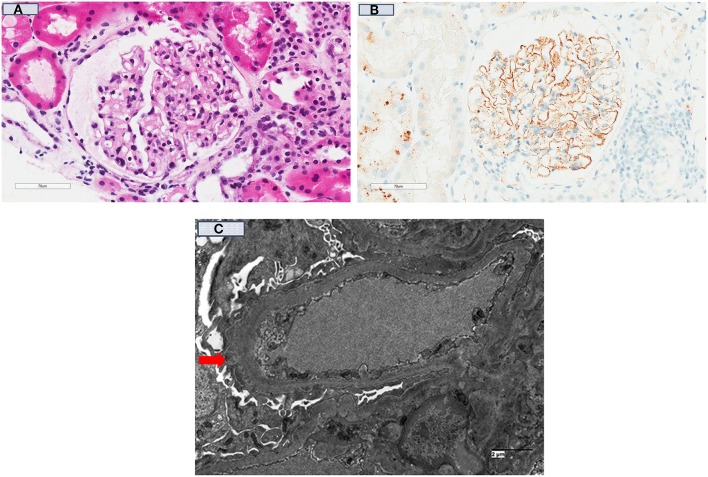
Kidney transplant recipient who had developed recurrent idiopathic membranous glomerulonephritis 3-months post-transplant. **(A)** [H & E (40x)]: Kidney allograft biopsy showed a glomerulus with no significant changes and no spikes were seen on silver stains; **(B)** showed diffuse positive granular capillary loop staining with C4d immuno-peroxidase stain; and **(C)** (Electron Microscopy) showed a capillary loop containing a few small subepithelial electron-dense deposits (red arrow) in keeping with the diagnosis of early recurrent membranous GN.

The rate of disease recurrence in patients with idiopathic membranous GN following kidney transplantation is between 30 and 50%, with the disparate detection rates reported in the studies influenced by the characteristics of the cohort (e.g., those with high titres of circulating anti-PLA2R autoantibody have a greater risk of recurrent disease), follow-up period and dissimilar biopsy practices (Table 1) (4, 158). Even though the circulating levels of anti-PLA2R autoantibody tend to decline post-transplant (adsorption into the allograft or the effect of immunosuppression), there is a direct relationship between the titer level and risk of disease recurrence post-transplant. The positive predictive value of pre-transplant anti-PLA2R antibodies for disease recurrence is 83%, but the risk of recurrence in those with idiopathic membranous GN not attributed to anti-PLA2R antibody remains unknown (45, 154). Nevertheless, the diagnostic threshold of anti-PLA2R antibody in defining the risk of disease recurrence remains poorly defined. The utility of monitoring anti-PLA2R antibody post-transplant remains unclear, but should be considered in those with high pre-transplant circulating levels of anti-PLA2R antibody, in those with early disease recurrence (to predict disease progression), to determine response to treatment and to differentiate disease recurrence from *de novo* membranous GN or other causes of proteinuria (4, 159). The prognostic significance of other biomarkers (antibody to other auto-antigens) shown in Table 2 remains unknown. Figures 3A–C show the light microscopy and ultrastructural features of a kidney transplant recipient who had developed early recurrence of idiopathic membranous GN within a month post-transplant. The transplant allograft biopsy showed features of early membranous GN with a few small subepithelial deposits.

**Figure 4:**
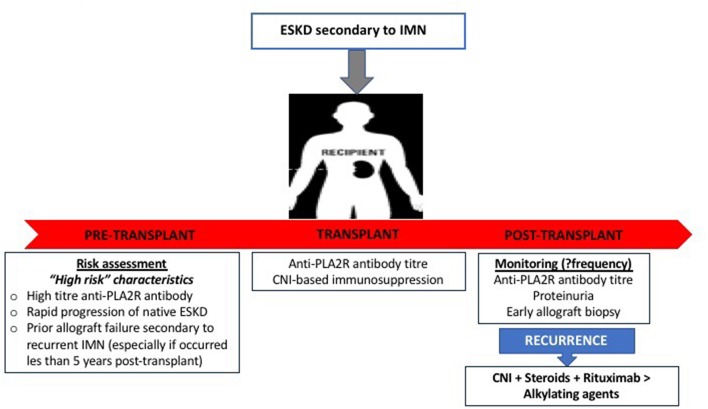
Practical approach to the pre- and post-kidney transplant risk assessment and management of patients with end-stage kidney disease secondary to idiopathic membranous glomerulonephritis.

The treatment of disease recurrence is largely extrapolated from treatment in the general population and typically includes a combination of anti-proteinuric agents, corticosteroids, alkylating agents, CNI, and rituximab. Given that kidney transplant recipients are likely to be maintained on corticosteroids and CNI, most clinicians would advocate continuing (or changing from mTOR inhibitor-based regimen to) CNI and consider the addition of anti-CD20 antibodies rather than introducing alkylating agents to avoid over-immunosuppression resulting in severe infective complications. Rituximab is an effective treatment for native and recurrent membranous GN in the allograft, with up to 80% achieving complete or partial remission with the use of rituximab for early disease recurrence post-transplant (160–163). A personalized approach of prescribing rituximab only to patients with positive anti-PLA2R antibody-associated idiopathic membranous GN in the native kidneys has been suggested (164, 165), but a similar approach has not been advocated for disease recurrence. The decision and timing of initiating specific treatment, in addition to anti-proteinuric treatment for patients with recurrent idiopathic membranous GN remains unknown, as many patients may have subclinical histological recurrence (particularly those with pre-transplant circulating anti-PLA2R antibody) or the proteinuria may be attributed to other concurrent diseases (e.g., transplant glomerulopathy). A single case of complete clinical remission with bortezomib has been described for rituximab-resistant recurrent membranous GN post-transplant, suggesting that depletion of plasma cells (in addition to B cells) may be needed in refractory cases (166). There is currently insufficient information as to the pre-emptive use of rituximab for patients with idiopathic membranous GN and high pre-transplant levels of anti-PLA2R antibody, but this (or early initiation post-transplant) can be considered in those with detectable high levels of anti-PLA2R antibody with prior allograft failure from recurrent membranous GN or have persistent high or increasing levels of circulating anti-PLA2R antibody post-transplant with early histological recurrence. The higher relative risk of allograft failure (up to 50% at 10-years of follow-up) following disease recurrence is a point of concern but must be taken into context the varying rates of disease recurrence, potential ascertainment bias of attributing chronic allograft failure from recurrent disease and the competing risk of other causes of allograft failure and death with a functioning graft (5, 167); and more detailed analysis of these cases to identify potentially modifiable factors that may explain those at risk of allograft failure following disease recurrence are required (e.g., ineffective treatment or delayed institution of treatment). A practical approach post-transplant in patients with idiopathic membranous GN is shown in Figure 4 and Table 3.

### Secondary GN

In contrast to ESKD attributed to primary GN, patients with secondary GN subtypes attributed to systemic diseases [such as atypical hemolytic uremic syndrome (aHUS), systemic lupus erythematosus (SLE), anti-glomerular basement membrane (GBM) disease, and crescentic GN (e.g., from systemic vasculitis)] may experience GN recurrence after kidney transplantation, but these relapses often occur later post-transplant and infrequently lead to allograft failure (8). In patients with anti-neutrophil cytoplasmic antibody (ANCA)-associated vasculitis, the relapse rate (often extra-renal complications) has been reported at 0.02 per patient-years, with no consistent association demonstrated between ANCA subtypes or disease severity prior to transplantation and disease recurrence occurring post-transplant (168–170). The incidence and outcome of patients with recurrent lupus nephritis post-kidney transplantation are unclear, with the majority of the reports from single-center and registry studies. The reported incidence of recurrent lupus nephritis varies between 0 and 44% (171), with patients who had experienced recurrent lupus nephritis at a greater risk of allograft failure and mortality (172–175). However, this association remains inconsistent (176). In patients with anti-GBM disease, disease recurrence after kidney transplant is <5% in the era of modern immunosuppression, and allograft failure from disease recurrence exceedingly rare (177, 178). Prior to the availability of the C5-inhibitor eculizumab (Soliris^®^), the risk of disease recurrence following transplantation of patients with aHUS was up to 80%, with a substantial proportion of patients losing their allografts from recurrent disease within the first year post-transplant and therefore, kidney transplantation was considered a contraindication for patients with aHUS (179). With prophylactic use of eculizumab, kidney transplantation appeared safe with excellent allograft outcome, even in those with pathogenic mutations known to be associated with a high risk of disease recurrence (180–182). There have been reports of successful live donor kidney transplantation without the use of prophylactic eculizumab but this is generally not recommended or undertaken with extreme caution in patients with certain genetic mutations (e.g., membrane-associated complement regulator membrane cofactor protein mutation) and frequent post-transplant monitoring for disease recurrence (183, 184).

### Presumed or Advanced GN

In patients with ESKD where the underlying etiology of the cause of ESKD is uncertain because a biopsy was not undertaken (but with clinical suspicion) or were non-diagnostic with non-specific histological features of interstitial fibrosis and glomerulosclerosis, the incidence of allograft failure attributed to GN is extremely low. Data from the ANZDATA registry showed that 5% of death censored allograft failure of kidney transplant recipients with presumed/advanced GN as cause of ESKD was attributed to GN, compared to over 30% in those with high-risk primary GN subtypes. In this study, almost 90% of the allograft failures from GN were attributed to FSGS, membranous GN and IgA nephropathy, but it is unknown whether these GNs represent recurrent or *de novo* GN (185). Similar findings have been corroborated in a large Canadian population cohort study where the incidence of post-transplant biopsy-proven GN was diagnosed in 5.7% (9 of 159 patients) of patients with presumed GN (cumulative probability of 11.8% at 15 years post-transplant), with 8 of 9 post-transplant GN cases attributed to FSGS and IgA nephropathy (9).

### *De novo* GN

The true prevalence of *de novo* GN in kidney transplant recipients remains unknown, but is associated with significantly reduced allograft survival compared to those without *de novo* glomerular disease. The incidence of *de novo* GN after kidney transplant varies between 4 and 20%, with FSGS, IgA nephropathy, membranous GN, and MPGN being the most common *de novo* GN subtypes. There are difficulties in identifying and confirming the presence of *de novo* GN post-transplant because the cause of native ESKD is often uncertain (kidney biopsies were often not undertaken or were non-diagnostic), the presence of GN in the donor kidney may not be known (particularly in the absence of pre-implantation biopsy), variations in allograft biopsy practices and differences in the histopathological evaluation of allograft biopsies where immunofluorescence and electron microscopy of biopsies may not be routinely performed (8, 186). In a Canadian cohort study, the incidence of *de novo* GN occurred in 3.4 and 3.6% of those with primary ESKD from GN and those with ESKD from non-GN causes, corresponding to cumulative incidences of 9.6 and 10.5% at 15 years, respectively. In patients whose ESKD was attributed to non-GN causes and had developed *de novo* GN, over 95% of cases were FSGS (the most common form of *de novo* GN, 11 of 26 [42%] cases), IgA nephropathy (7 of 26 [27%]), membranous GN (4 of 26 [15%]), and MPGN (3 of 26 [12%]) (9). In this study, the risk of allograft failure was over 7-times greater among recipients who have developed *de novo* GN, compared to those without disease.

Table 4 shows the subtypes of *de novo* GN that have been reported, outlining the differences in clinical and histological characteristics of *de novo* vs. native (or recurrent) GN, as well as potential treatment options and outcomes. The presentations of those with *de novo* GN are similar to those with primary GN subtypes, ranging from asymptomatic urinary or biochemical abnormalities to overt symptoms and signs of GN with nephrotic syndrome and renal dysfunction. As way of an example, there are several risk factors that may predispose to the development of *de novo* FSGS, particularly those associated with a reduction in nephron mass (resulting in compensatory hyperfiltration of remaining nephrons such as diabetes, hypertension, BK viral infection, CNI therapy, and rejection) or the introduction of sirolimus (through the effects on podocyte integrity) (96, 186, 225–227). Consequently, it is often difficult to differentiate secondary forms of FSGS (developing post-transplant) from “actual” *de novo* non-collapsing GN and may to some extent explain why most cases of *de novo* FSGS tend to occur later post-transplant (compared to the recurrence of primary FSGS). Irrespective of the nature of *de novo* FSGS, the long-term allograft outcome is relatively poor, particularly in the presence of interstitial fibrosis/tubular atrophy with 5-year allograft survival of <50% after diagnosis (191, 228). Given the heterogeneity of the disease process, the treatment of *de novo* FSGS predominantly revolves around adequate anti-proteinuric treatment and the removal of the offending agents/factors where possible, but more aggressive therapy (similar to disease recurrence) may be considered, although there are no data to support this approach. Re-transplantation following allograft failure from *de novo* FSGS can be considered, but clinicians should attempt to establish and exclude or avoid potential causative factors that had resulted in the development of *de novo* FSGS.

**Table 4:** Characteristics and differences between *de novo* glomerulonephritis compared to recurrence of primary glomerulonephritis after kidney transplantation.

**GN subtypes**	**Clinical characteristics**	**Differences to primary GN recurrence**	**Treatment options**	**Outcome/retransplantation**
**“Commonly recurring”** ***de novo*** **GN subtypes**				
IgA nephropathy	Asymptomatic hematuria to rapidly progressive GN. Possibility of donor-transmitted IgA nephropathy (22, 54, 187, 188)	None reported	No specific treatment, similar options to primary disease	Generally favorable unless crescentic GN, re-transplantation possible
FSGS (non-collapsing)	Most common *de novo* GN subtype (may be up to 20%), possibly secondary FSGS (virus, diabetes, drugs such as CNI and mTOR-inhibitors) (9, 96, 189, 190)	Occurs later post-transplant compared to recurrent FSGS	Identify, eliminate and treat “offending” agents/insults if present	Prognosis poor, 40% 5-year renal allograft survival (especially in presence of CAN) (191, 192), re-transplantation possible
Membranous GN	Incidence 2%, may be secondary (e.g., antibody-mediated rejection, viral hepatitis) (193, 194)	Potential differences in histology/IF findings: IgG1 staining more dominant (vs. IgG4 in recurrent disease) and likely to exhibit positive glomerular staining for phospholipase A2 receptor (195–198)	Similar to idiopathic type (199)	Unclear, possible higher risk of allograft failure. Re-transplantation possible
MPGN	Incidence up to 3%, with secondary Ig-mediated MPGN related to HCV infection, TMA, rejection, and other systemic diseases (186, 200, 201)	*De novo* C3-GN rare with 1 case-report (recurrence common) (202)	Identify, eliminate and treat “offending” agents/insults if present. No effective treatment	High risk of allograft failure, caution if considering re-transplantation
**“Rarely recurring” or “distinct”** ***de novo*** **GN subtypes**				
Minimal change GN	Typically early-onset, nephrotic syndrome. Possibly related to immunosuppressive agents (203–205)	Mild light microscopy abnormalities	Steroid-responsive	Excellent
Collapsing GN	Variant of FSGS, <1% prevalence. Nephrotic syndrome and allograft dysfunction (206–210)	Possible viral etiology and related to the presence of antibodies to angiotensin II type 1 receptor	None effective	Poor prognosis, majority with allograft failure
Fibrillary GN	Case reports, progressed to allograft failure (211)	Higher risk of recurrence for primary disease (50% recurrence, <20% allograft failure from recurrence) (212, 213)	None effective	Unknown, re-transplantation possible
Immunotactoid GN	Case report (x1), possible reversible association with CMV (214)	Variable recurrence rate for primary disease and potentially responsive to cytotoxics, plasmapheresis, or rituximab (215, 216)	Unknown	Potentially reversible if related to CMV
**“Systemic” or disease-specific** ***de novo*** **GN**				
Alport syndrome → anti-GBM disease	Uncommon occurrence in patients with Alport syndrome, with 3–5% developed anti-GBM disease (217–220)	–	Treatment as per primary anti-GBM disease	Allograft and patient survivals similar to other causes of ESKD. Re-transplantation possible
Pauci-immune GN (ANCA-positive or negative)	Rare cases reported, unlikely related to kidney transplantation (221–224)	Unknown	Treatment as per primary disease	Generally poor prognosis, unknown risk of re-transplantation

*GN, glomerulonephritis; FSGS, focal segmental glomerulosclerosis; MPGN, membranoproliferative GN; Ig, immunoglobulin; GBM, glomerular basement membrane; ESKD, end-stage kidney disease; ANCA, anti-neutrophilic cytoplasmic autoantibody; CMV, cytomegalovirus; HCV, hepatitis C virus; TMA, thrombotic microangiopathy; CAN, chronic allograft nephropathy; mTOR, mammalian target of rapamycin*.

The incidence of *de novo* IgA nephropathy is likely to be underestimated, with asymptomatic IgA deposition not infrequently found (detected incidentally in protocol or indication biopsies or potentially donor-derived asymptomatic disease detected on pre-implantation biopsies) (186, 187). However, presentation with macroscopic hematuria is unusual. The prognosis of those with *de novo* IgA nephropathy is usually relatively benign with treatment predominantly focuses on anti-proteinuric and/or anti-hypertensive therapy (188). Patients who have developed crescentic *de novo* IgA nephropathy tend to have a poorer prognosis (22), but the efficacy of more aggressive treatment with steroids and alkylating agent remains unknown. There is insufficient data to determine whether the clinical course of those with recurrent or *de novo* IgA nephropathy is dissimilar, but re-transplantation of patients with either disease is possible. *De novo* membranous GN and MPGN are much less common, and the reported cases primarily related to secondary causes including viral infections, rejection, autoimmune disease, CNI and thrombotic microangiopathy (115, 193, 229). The onset of *de novo* membranous GN or MPGN tend to occur later post-transplant (compared to recurrent disease), with symptoms ranging from asymptomatic detection of mild proteinuria and incidental biopsy findings to nephrotic range proteinuria and rapidly progressive GN (with acute allograft deterioration), but these are often indistinguishable compared to the timing, presentations, and clinical course of those with recurrent diseases (50). For membranous GN, the pattern of Ig staining or glomerular staining for PLA2R may help to differentiate *de novo* from recurrent disease. In patients with recurrent disease, IgG1 staining in capillary loop deposits was dominant/co-dominant (*n* = 7 cases), whereas, IgG4 staining in capillary loop deposits was dominant/co-dominant (*n* = 2 cases) in *de novo* disease (195). In another small study of 24 patients with recurrent (*n* = 12) or *de novo* membranous GN (*n* = 12), glomerular staining for PLA2R was more common in those with recurrent disease (83% cases, sensitivity and specificity of 83% [95%CI 51–97%] and 92% [95%CI 60–100%], respectively) compared to *de novo* disease (17%), and suggest that injury to the podocytes occurring post-transplantation triggering the release of podocyte antigens may induce the formation of auto-antibodies and deposition of subepithelial immune complexes (193, 196). These findings will need to be validated in larger cohorts. Nevertheless, in all patients with *de novo* membranous GN and MPGN, a careful histological examination for potential secondary causes or contributing factors should be undertaken, which will have clinical implication when considering treatment options and future re-transplantation potential for these patients.

Clinicians should be aware of the need to exclude secondary causes in recipients who have developed *de novo* GN, which is critical when considering treatment options and re-transplant potential (following allograft failure). Nonetheless, the systematic approach to the investigations and subsequent management of kidney transplant recipients with *de novo* GN is similar to that of patients presenting with GN without a kidney transplant. However, the data informing the clinical and pathological differences between recurrent compared to *de novo* GN remains limited and therefore the current understanding of the epidemiology, pathogenesis and outcomes of *de novo* GN is likely to evolve with the availability of future studies.

## Conclusion

Despite the advances in the understanding of the epidemiology, pathogenesis, and classification of primary and recurrent GN after kidney transplantation, considerable uncertainty remains in the approach and treatment of post-transplant GN recurrence (230). It is therefore imperative to consider the establishment of a global GN registry, focusing on data collection on the clinical characteristics, histological features, treatment, and outcome of patients who have developed post-transplant GN, particularly those with high risk GN subtypes. The Post-TrANsplant GlOmerular Disease (TANGO) study, an observational, multicenter cohort study was initiated in 2017 by researchers across Europe, North American, and South America, with the goal of collecting patient-level data regarding the risk factor, trajectory of disease activity, and responses to treatment; to develop a bio-repository of human specimens including blood, cell, and tissue samples; and the opportunity to undertake collaborative studies and clinical intervention trials to improve the management and clinical outcomes of these patients (231). Even though the clinical care of patients with recurrent or *de novo* GN remains challenging, it is an exciting time for the integration of translational GN-related research to novel developments in the understanding and treatment of GN, with the ultimate goal of establishing personalized treatment by effectively tailoring specific treatment strategies to patients with the various types of recurrent or *de novo* GN.

## Disclosure

WL was supported by a Clinical Research Fellowship from the Raine Foundation (University of Western Australia and Health Department of Western Australia) and Jacquot Research Foundation (Royal Australasian College of Physicians). GW was supported by a National Health and Medical Research Council Career Development Fellowship.

## Author Contributions

WL designed the outline of the manuscript and prepared the tables/figures. MS provided pathology slides. All authors wrote the manuscript.

### Conflict of Interest Statement

The authors declare that the research was conducted in the absence of any commercial or financial relationships that could be construed as a potential conflict of interest.
